# Comparative Analysis of the Host Response in a Rat Model of Deep-Partial and Full-Thickness Burn Wounds With *Pseudomonas aeruginosa* Infection

**DOI:** 10.3389/fcimb.2019.00466

**Published:** 2020-01-10

**Authors:** Alan J. Weaver, Kenneth S. Brandenburg, Brian W. Smith, Kai P. Leung

**Affiliations:** ^1^Department of Dental and Craniofacial Trauma Research, United States Army Institute of Surgical Research, JBSA Fort Sam Houston, San Antonio, TX, United States; ^2^Research Support Division, United States Army Institute of Surgical Research, JBSA Fort Sam Houston, San Antonio, TX, United States

**Keywords:** *Pseudomonas aeruginosa*, DAMPs, burn, cytokine, infection, biofilm

## Abstract

Burn wound injury affects soldiers and civilians alike, often resulting in a dynamic, but un-orchestrated, host response that can lead to infection, scarring, and potentially death. To mitigate these factors, it is important to have a clinically relevant model of burn wound infection that can be utilized for advancing burn wound treatments. Our previous reports have demonstrated the ability of *Pseudomonas aeruginosa* to generate a biofilm infection within a modified Walker-Mason rat burn model of deep-partial (DPT) and full-thickness (FT) burn wounds (10% total body surface area) in male Sprague-Dawley rats (350–450 g). Here, we further define this model with respect to the host response when challenged with *P. aeruginosa* infection between the two burn types. Following burn injury and immediate surface exposure to *P. aeruginosa*, inflammation at the local and systemic levels were monitored for an 11 days period. Compared to burn-only groups, infection with *P. aeruginosa* further promoted local inflammation in both DPT and FT burn wounds, which was evident by enhanced cellular influx (including neutrophils and monocytes), increased levels of several pro-inflammatory cytokines (IL-1β, IL-6, GRO/KC, andMIP-1α), and reduced IL-10. Systemically, only minor changes were seen in circulating white blood cells and cytokines; however, increases in high mobility group box-1 (HMGB-1) and hyaluronan, as well as decreases in fibronectin were noted particularly in FT burns. Compared to the burn-only group, *P. aeruginosa* infection resulted in sustained and/or higher levels of HMGB-1 and hyaluronan. Combined with our previous work that defined the burn depth and development of *P. aeruginosa* biofilms within the wound, this study further establishes this model by defining the host response to the burn and biofilm-infection. Furthermore, this characterization shows several similarities to what is clinically seen and establishes this model for future use in the development and testing of novel therapeutics for burn wound treatment at home and on the battlefield.

## Introduction

Burn injury compromises the primary barrier of the host, the skin, which immediately places the host at risk for infection (Church et al., 2006; Kennedy et al., 2010; Lopez et al., 2017). This poses a particular threat to soldiers, who have limited access to immediate medical care at the point of injury, let alone a sterile surgical environment. Furthermore, research has shown similar trends in both combat and non-combat military situations with regard to burn area and location, which are typically 10% total body surface area (TBSA) and located around the hands and face (Kauvar et al., 2006). These trends were also noted to occur in civilian life (Wolf et al., 2006). In broader terms, the World Health Organization estimates that 180,000 deaths are caused by burns on a yearly basis, with non-fatal burn injuries being a leading cause of morbidity (Organization, 2018). In 2016, an estimated 486,000 burn injuries required medical treatment within the United States alone, with 40,000 cases requiring hospitalization (Association, 2018). While hospitalization can be necessary, it may place the patient at a greater risk of infection, formation of biofilms, and even sepsis, ultimately leading to greater mortality, especially for patients suffering with greater than a 40% TBSA burn injury (Church et al., 2006; Lopez et al., 2017). These aspects points to the need for continued research efforts in burn therapeutics for both military and civilian application, as well as appropriate pre-clinical models for testing potential therapeutics.

The microenvironment of a burn wound is well-suited for various microbes to take up residence. *Pseudomonas aeruginosa*, a gram-negative bacterium, is one of the most common pathogens to infect burn patients and is well-known for its capability to form biofilms within several types of wounds (Bodey et al., 1983; Santucci et al., 2003; Tredget et al., 2004; James et al., 2008). Acute stages of *P. aeruginosa* infection can result in an overactive immune response that results in significant inflammation of the wound. Infection stimulates the recruitment of several immune cells, with neutrophils being the primary responders. While recruitment of neutrophils is desired for clearance of unwanted microbes, *P. aeruginosa* can stimulate an over influx of neutrophils as part of its defensive mechanisms against the host. In particular, the Type 3 secretion system of *P. aeruginosa* has several effectors (including ExoS, ExoT, and ExoU) that assist the bacterium in neutrophil evasion (Diaz et al., 2008; Sun et al., 2012; Alhede et al., 2014; Lin and Kazmierczak, 2017). Furthermore, the activities of neutrophils are further thwarted in the wound by biofilm generation, which is resistant to neutrophil-mediated killing (Jesaitis et al., 2003; Bjarnsholt et al., 2005). This dysfunction in neutrophil activity generates greater inflammation and a non-resolving host response (continuous inflammation) to the infection (Cox et al., 1995; Alhede et al., 2014), contradicting the normal course of healing which requires dampening of pro-inflammatory cytokines, such as IL-1β, IL-6, and IL-8, and increased levels of anti-inflammatory cytokines, like IL-10 (Ding et al., 2015).

In our previous work, we introduced a modified Walker-Mason scald model for inducing deep-partial thickness (DPT) and full-thickness (FT) burn wounds, followed by infection with a clinical isolate of *P. aeruginosa* (strain 12-4-4) (Walker et al., 1964; Karna et al., 2016; Brandenburg et al., 2019a,b). This rat model provides consistent burn depths and generates a *P. aeruginosa* biofilm infection that could be monitored over an 11-days period. While the previous work focused on the invasive nature of *P. aeruginosa*, the present work looked to understand the host response to both the injury and biofilm infection in this model in relation to clinical findings. Systemic changes were minimal; however, a robust local inflammatory response was seen following burn injury, which was further enhanced by the presence of *P. aeruginosa*. Large influx of neutrophils was noted at the site of injury, which resulted in greater tissue damage and ultimately elevated damaged-associated molecular patterns (DAMPs) within the circulatory system that was relative to burn injury and infection. These results, coupled with our previous findings, provide a baseline for future use of this model in the testing of novel therapeutics focused on reduction in inflammation and biofilm infection in order to promote greater wound healing.

## Materials and Methods

### Animal Ethics Statement

All research was conducted in compliance with the Animal Welfare Act, the implementing Animal Welfare Regulations, and the principles of the Guide for the Care and Use of Laboratory Animals, National Research Council. The facility's U.S. Army Institute of Surgical Research Institutional Animal Care and Use Committee approved all research conducted in this study under protocols A16-035 (DPT burn) and A16-047 (FT burn) on May 31, 2016, and September 15, 2016, respectively. The facility where this research was conducted is fully accredited by AAALAC.

### Overview of Scald Burn Model

A DPT or FT scald burn was inflicted on the dorsum of anesthetized rats by methods previously described (Brandenburg et al., 2019b). In brief, the day preceding the burn, male Sprague-Dawley rats weighing 350–450 g were anesthetized with isoflurane (Forane, Baxter Healthcare Corporation, Deerfield, IL, USA) prior to the dorsum being shaved and depilated with Nair (Church & Dwight Co., Inc., Ewing, NJ, USA). Following hair removal, rats were given a *subcutaneous* injection of Buprenorphine SR LAB (1.2 mg/kg, Zoopharm Pharmacy, Windsor, CO, USA) for pain management.

On the day of the burn, rats were anesthetized with isoflurane, placed into the mold and administered a DPT or FT burn equating to 10% total body surface area (TBSA), based on Meeh's formula (Gilpin, 1996). Immediately following the burn, the wound surface was inoculated with 100 μL of 10^3^ or 10^4^ CFU/wound of *P. aeruginosa* (strain 12-4-4) suspended in phosphate buffered saline (PBS) (Karna et al., 2016). Control animals received the same burn injury, but received 100 μL of sterile PBS (i.e., no *P. aeruginosa*). Following inoculation, wounds were covered with a non-adherent interface dressing, followed by Tegaderm™ Film (3M Health Care, St. Paul, MN). Rats were placed in custom rat jackets (Brandenburg et al., 2019b) to prevent tampering with the wound throughout the study, and monitored over an 11 days period.

Animals were euthanized at post-operative days (POD) 1, 3, 7, and 11 for assessment of burn wound injury and infection. At each endpoint, animals were initially anesthetized with 100 mg/kg Ketamine HCl (Zetamine, MWI Veterinary Supply Co. Boise, ID) and 10 mg/kg Xylazine (Akorn Animal Health, Inc., Lake Forest, IL), utilizing isofluorane via nosecone to ensure depth of surgical anesthetic plane, as needed. Prior to intra-cardiac injection of Fatal-Plus® (Vortech Pharmaceuticals, Ltd., Dearborn, MI), blood was acquired by cardiac puncture. Following administration of Fatal-Plus®, euthanasia was confirmed by lack of cardiac movement, pulse, and breathing as specified by the *AVMA Guidelines for the Euthanasia of Animals: 2013 Edition* (Leary et al., 2013). Following euthanasia, wound tissue was isolated from the dorsum for bioburden, histology, and biochemical analysis. Total number of animals utilized per POD were *n* = 3 for burn-only controls and *n* = 6 for each inoculum of *P. aeruginosa*.

### Blood Collection and Processing

Blood was collected into serum separator (Vacuette® 454228P, Greiner Bio-One, Kremsmünster, Austria), EDTA-coated (Vacutainer® 367841, Becton Dickinson, Franklin Lakes, NJ, USA), and sodium citrate (Vacutainer® 363083, Becton Dickinson) tubes. Serum and citrate-plasma were collected per manufacturer's instructions. EDTA plasma samples were submitted for complete blood count analysis on an Abbott Cell-DYN® 3700 Blood Count Analyzer (Abbott Laboratories, Abbott Park, IL, USA), followed by plasma isolation per manufacturer's instructions. All serum and plasma was immediately frozen at −80°C until needed.

### Histopathological Assessment

Following excision, cross-sections of the burn wound tissue were immediately fixed in 10% buffered formalin in PBS (Fisher Scientific, Kalamazoo, MI, USA) for at least 48 h. Tissue was processed for routine embedding in paraffin wax, followed by sectioning (4–5 μm) and staining with hematoxylin-eosin (H&E). H&E slides were assessed by a trained veterinary pathologist using a semi-quantitative analysis for inflammation based on infiltration of inflammatory cells. Inflammation was described using the following scale of infiltration: zero is none, one is 0–10%, two is 10–25%, three is 35–50%, and four is >50%.

### Damage-Associated Molecular Patterns (DAMPs) Analysis

#### High Mobility Group Box-1

Collected sera was analyzed for high mobility group box-1 (HMGB-1) protein using a Rat HMGB-1 ELISA kit (#50155150, Fischer HealthCare, Inc.). Samples were assayed undiluted on a DSX® Automated ELISA System (Dynex Technologies, Inc., Chantilly, VA, USA) per kit manufacturer's instructions.

#### Hyaluronan and Fibronectin

EDTA collected plasma was analyzed for the extracellular matrix proteins, hyaluronan (HYL) and fibronectin (FBN). For HYL, plasma was diluted 1:4 with provided diluent prior to being assayed with a Hyaluronan Quantikine ELISA Kit (DHYLA0, R&D Systems Inc., Minneapolis, MN, USA). Due to the natural presence of FBN, plasma was diluted 1:40,000 with provided diluent prior to being assayed with a Rat Fibronectin ELISA kit (ab108850, Abcam, Cambridge, UK). Assays were performed on a DSX® Automated ELISA System per each kits manufacturer's instructions.

### Cytokine and Chemokine Panel

Systemic and local cytokines/chemokines were assessed using a Bio-Plex Pro™ Rat Cytokine 23-Plex (#12005641, Bio-Rad, Hercules, CA, USA) kit, which was analyzed on a Bio-Plex™ 200 System (Bio-Rad). For systemic cytokines/chemokines, sera was diluted 1:4 with provided sample diluent and then assayed per manufacturer's instructions. Local cytokines/chemokines were assayed from the 7-mm biopsy punches collected from the wound bed. Samples were prepared using a Bio-Plex® Cell Lysis Kit (#171304011, Bio-Rad). In brief, two biopsy punches were initially pulverized under liquid nitrogen using a Bessman Tissue Pulverizer (Spectrum, Inc., Rancho Dominguez, CA) and then suspended in 500 μL of prepared lysis buffer prior to homogenization. Samples were homogenized with an IKA T10 basic Ultra Turrax tissue homogenizer (IKA Works, Inc., Wilmington, NC, USA) followed by one freeze-thaw cycle. Upon defrosting, samples were sonicated for a total of 30 s (5 s intervals, with icing in between) with a Sonic Dismembrator Model 100 (Fisher Scientific) and then centrifuged at 12,000 × g for 5 min at 4°C to isolate supernatants. Protein concentration of supernatants was quantified using a Pierce™ BCA Protein Assay Kit (#23227, Thermo Fisher Scientific). For cytokine/chemokine analysis, samples were normalized to 900 μg/mL protein and then assayed per manufacturer's instructions. All washes were performed using a Bio-Plex Pro™ Wash Station. Systemic and local cytokine/chemokines were quantified using the BioPlex Manager 6.1 software and normalized to control for lot differences.

### Statistical Analysis

All statistical analysis was performed in GraphPad Prism 8 (GraphPad Software, San Diego, CA). Values are presented as median with interquartile range, unless otherwise stated. Significant differences were established based on non-parametric analysis followed by Dunn's multiple comparison post-test, unless otherwise stated. Hashtags (#) define differences relative to DPT burn-only group. Asterisks (^*^) define differences relative to FT burn-only group.

## Results

### Pathological Assessment

**Figure 1:**
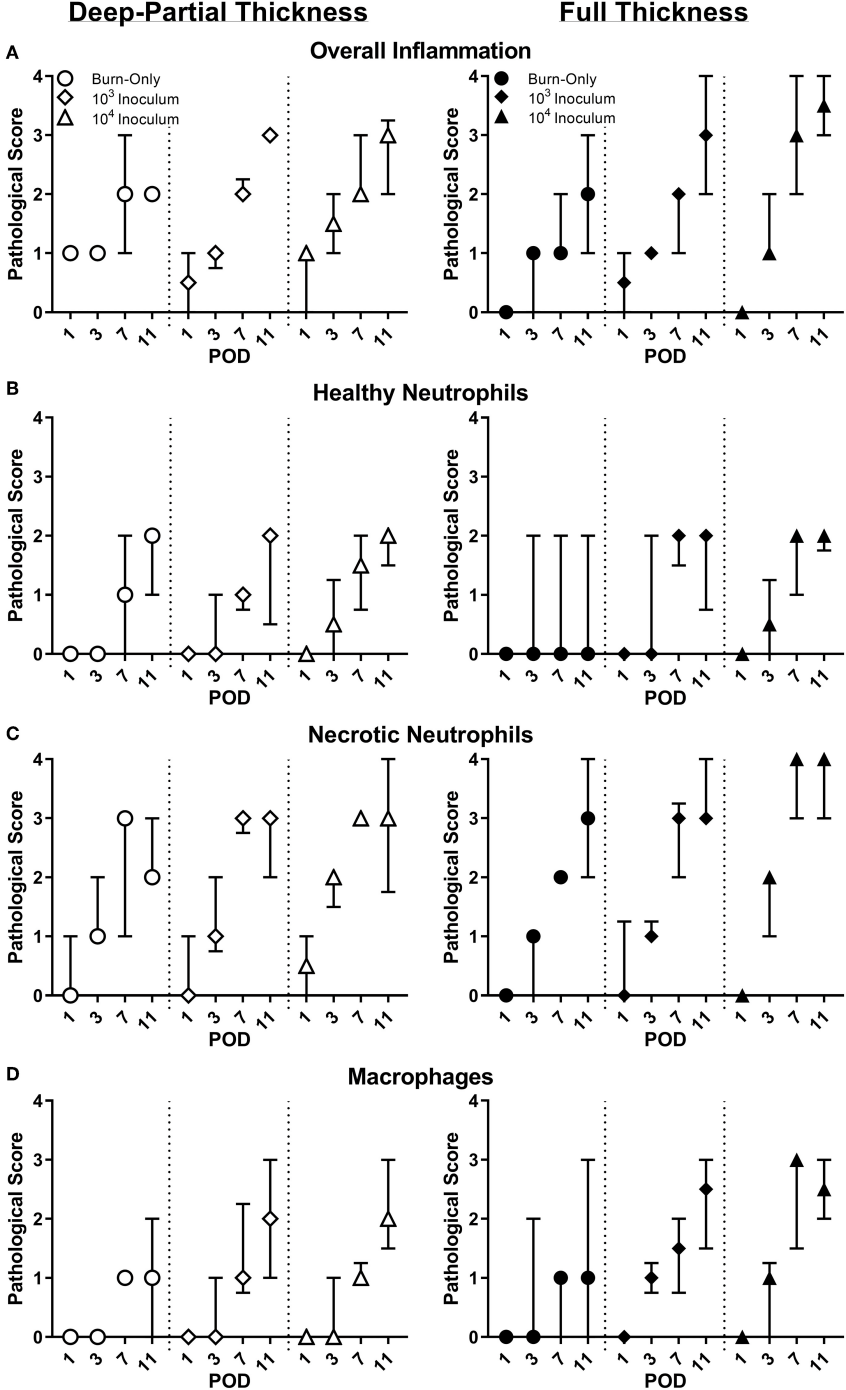
Pathological assessment of DPT & FT burn wounds. Overall inflammation **(A)** was elevated from the burn wound injury; however, infected wounds saw a greater inflammation by POD 11. Compared to DPT injuries, *P. aeruginosa* also triggered faster and/or greater influx of neutrophils **(B,C)** and macrophages **(D)** into FT burn wounds following a 10^4^ CFU/wound inoculation.

By POD 11, the overall inflammation the burn alone was similar for both burn scenarios. However, there was a delayed response in inflammation for FT burns at POD 1 compared to DPT (Figure 1A). Inflammation and cell infiltration were more elevated by the presence of *P. aeruginosa*, in a dose dependent fashion. Neutrophils provided a particularly strong response to the burn trauma; however, the majority of these cells were noted to be degenerate and/or necrotic (Figures 1B,C). This was especially true in the presence of *P. aeruginosa*, where these cells were present earlier on and/or higher in number, particularly in the FT burn wound. Macrophages showed similar response between the burn-only groups, and followed a similar trend with respect to necrotic neutrophils in the presence of *P. aeruginosa*, being more elevated by POD 7 (Figure 1D).

### Local Cytokines

**Figure 2:**
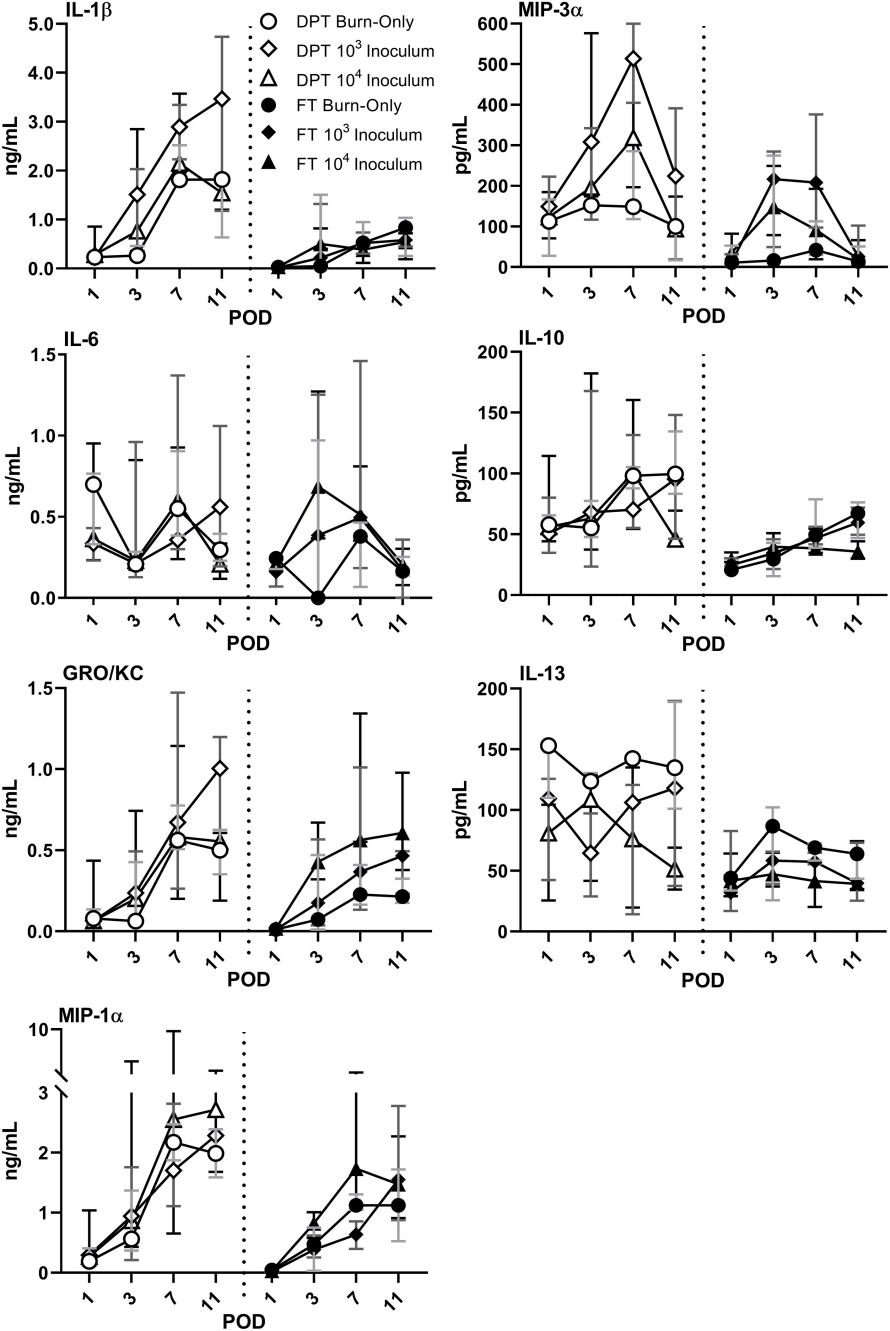
Local cytokine & chemokine profile following DPT & FT injury. Burn injury alone induced elevations in both pro- and anti-inflammatory cytokines, with the exception of MIP-3α in FT burn wounds. Relative to burn-only groups, infection with *P. aeruginosa* induced greater elevations in some pro-inflammatory cytokines, as well as MIP-3α in both DPT and FT burns. However, anti-inflammatory cytokines IL-10 and IL-13 were inhibited by the infection. Gray, dark gray, and black bars represent the interquartile ranges of burn-only, 10^3^ inoculum, and 10^4^ inoculum, respectively.

Burn injury alone resulted in elevations of several cytokines and chemokines (Figure 2 and Supplemental Figure 1). DPT and FT resulted in continuous elevations of pro-inflammatory cytokines IL-1β, GRO-KC, and MIP-1α, as well as anti-inflammatory cytokine IL-10 (Figure 2). IL-6 response more varied following DPT burns, particularly between infectious groups; however, IL-6 spiked at POD 3 and then rapidly declined in all groups in FT burn injuries. Anti-inflammatory cytokine IL-13 remained fairly constant between burn-only groups, albeit slightly more elevated in DPT than FT burn injuries. *P. aeruginosa* caused increases of pro-inflammatory cytokines GROK-KC and MIP-1α, while also suppressed expression of both IL-10 and IL-13, relative to the DPT or FT burn-only groups. Furthermore, *P. aeruginosa* resulted in spikes in chemokine MIP-3α from PODs 3–7, which were not seen in the burn-only groups.

### Complete Blood Counts

**Figure 3:**
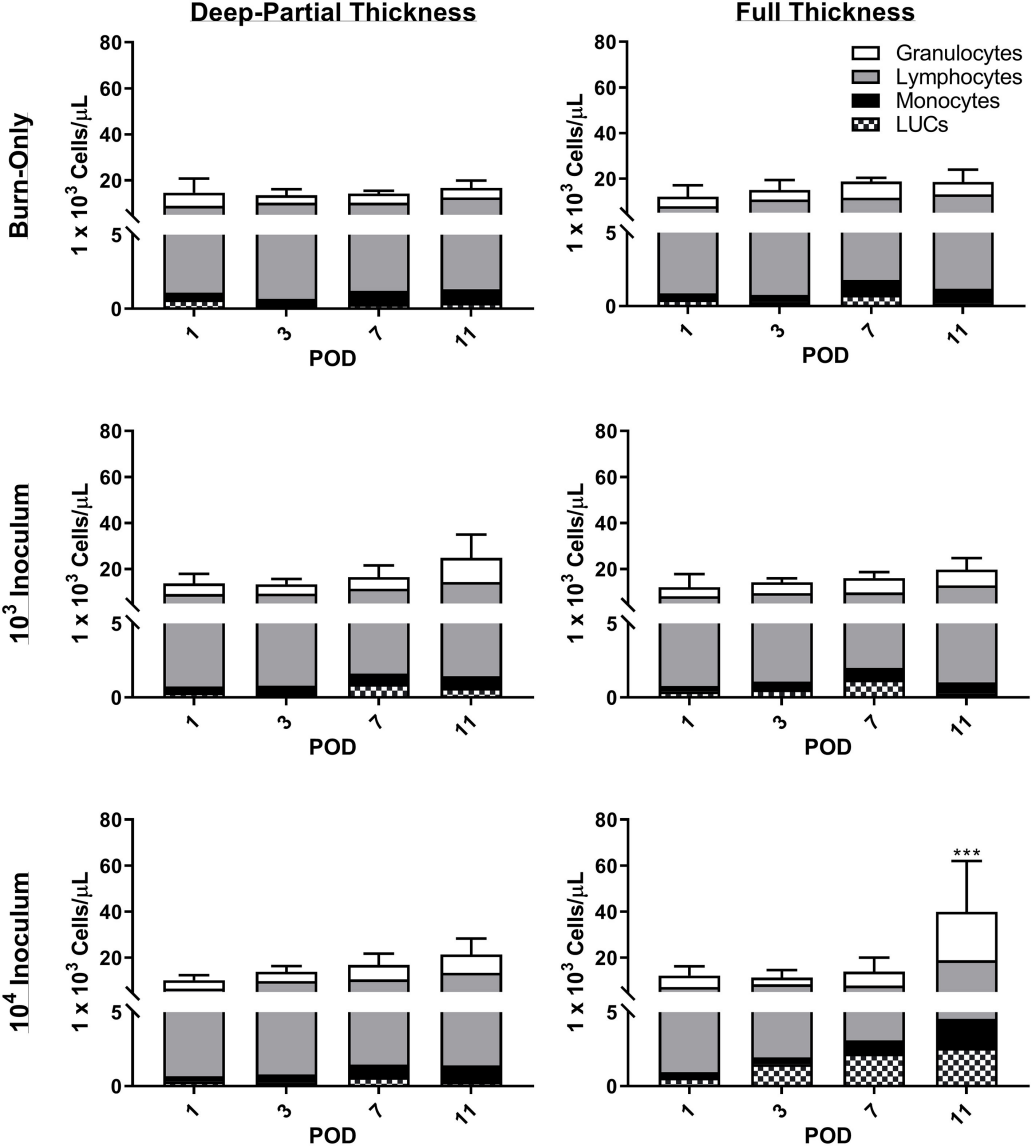
Complete blood count analysis reveals increases in white blood cells following DPT & FT injuries. Increased WBC were noted to occur over time in all cases and were related to overall increases in granulocytes, lymphocytes, and monocytes. *P. aeruginosa* infection showed slightly elevated WBC counts over the burn-only group, becoming significant in the 10^4^ CFU/wound group at POD 11 (***p < 0.001 relative to FT burn-only) based on 2-Way ANOVA with Bonferroni multiple comparison post-test. Bars represent average WBC count with standard deviations. Unassigned cells are represented as large unidentified cells (LUC).

Minor elevations in overall white blood cell (WBC) counts were seen in both DPT and FT burn wounds over the course of the entire study (Figure 3, Burn-Only). This global increase was attributed to elevations in granulocytes, monocytes, and lymphocytes. Although neither burn-only groups resulted in significant increases in leukocytes relative to sham (see Supplemental Table 1 for values), all infectious groups in DPT and the 10^4^ inoculum in FT were significantly elevated over sham by POD 11. Furthermore, only the 10^4^ inoculum in FT was significantly elevated over the FT burn-only group (*p* < 0.001). For absolute values and further breakdown of leukocytes, please refer to Supplemental Table 1.

### Circulating DAMPs and Cytokines

**Figure 4:**
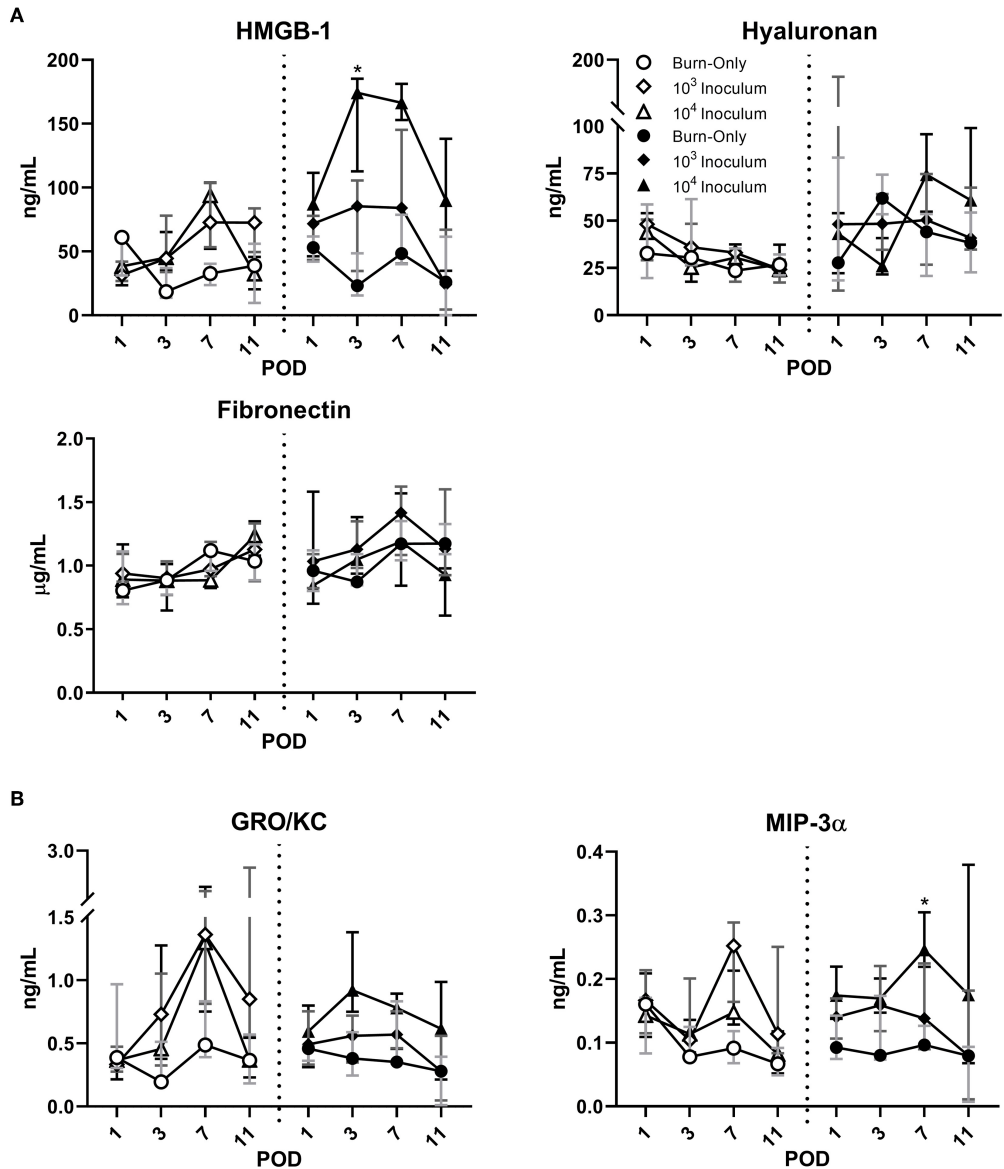
*P. aeruginosa* infection results in dynamic changes to circulating DAMPs **(A)** and chemokines **(B)**. HMGB-1 was elevated by DPT and FT burn injury at POD 1, but remained close to sham levels thereafter in the burn-only group. *P. aeruginosa* resulted in increased release of HMGB-1, particularly in FT burn wounds at most PODs. Extracellular matrix proteins, HYL and FBN, appeared to follow similar trends between all groups. Infection with *P. aeruginosa* may cause potential decreases in FBN, which were noted in both infected groups of FT by POD 11. While burn-only groups showed minimal changes in circulating chemokines, GRO-KC and MIP-3α were both elevated in the case of *P. aeruginosa* infection. Gray, dark gray, and black bars represent the interquartile ranges of burn-only, 10^3^ inoculum, and 10^4^ inoculum, respectively.

Compared to sham (Supplemental Figure 2), DPT and FT had elevated levels of HMGB-1 following the initial burn (Figure 4A). HYL showed only minor elevations after POD 1 in the case of FT, while FBN levels were seen to be initially low and then return to normal by POD 7 in both burn scenarios. Infection with *P. aeruginosa* resulted in increased levels of HMGB-1 that peaked at POD 7 in DPT burn wounds. In FT burns the HMGB-1 response was even greater in the case of the 10^4^ inoculum. The extracellular matrix proteins, HYL and FBN, followed very similar trends to that of the burn-only groups in both cases. However, a slight decrease was noted in the case of FT injury for both *P. aeruginosa* inoculums at POD 11.

Burn alone did not result in any changes to the circulating cytokines for either DPT or FT injury (Figure 4B and Supplemental Figure 3). However, *P. aeruginosa* infection did result in elevations of GRO/KC and MIP-3α at various stages depending on burn depth. Within DPT wounds, GROK/KC was elevated particularly at POD 7 in both inoculum groups; however, in the case of FT, only the 10^4^ inoculum generated notable elevations around POD 3. MIP-3α appeared to follow a mild increase that was inoculum dependent, showing the greatest increases in the 10^4^ inoculum group. These changes were not as consistent or noticeable in the case of DPT burns.

## Discussion

Burn injury results in a disruption of the skin barrier, to which the host elicits a complex and dynamic response. The response to burns is typically not well-orchestrated, as the host cells are bombarded with both pro- and anti-inflammatory signals resulting in confusion, overstimulation, and ultimately a lack of wound healing (Finnerty et al., 2006). Research and clinical efforts to understand the effects of burn injury on the host have made it apparent that the response can be highly variable and is governed by several factors related to the injury itself, as well as the patient (Kim et al., 2012). Therefore, it is paramount in the development of a new model to understand not only the injury itself, but to also establish the clinical relevance of the host response to the injury. In this light, these investigations looked to initially establish the host response to DPT and FT burns with respect to each other, and to understand how these responses are altered in the presence of a *P. aeruginosa* biofilm-infection within the confines of this model.

A dynamic local and systemic response to the DPT and FT burn was seen, with the end result being a highly inflamed wound bed. We initially focused on the injury itself (i.e., burn-only controls) in order to provide a baseline understanding of the insult under these conditions. As expected, pathological scoring revealed slightly greater inflammation—neutrophil influx—following FT burns, albeit with reduced local cytokines in most cases. Our previous findings of elevated levels of MPO were confirmed by pathology reports of a large influx of neutrophils to the wound; however, the majority were noted to be degenerate/necrotic (Brandenburg et al., 2019a,b). This uncontrolled cell death is effective at killing foreign bodies; however, it is also non-specific, resulting in severe damage to surrounding tissue, increased inflammation, and impaired healing. It was also noted that local GRO-KC, a homolog to human chemokine IL-8, was continuously elevated, which would drive neutrophil influx further, causing further damage. Along with GRO-KC, several other pro-inflammatory markers were elevated, including IL-1β, MIP-1α/3α, and IL-6; as well as the anti-inflammatory cytokines, IL-10 and IL-13. GRO-KC (IL-8), IL-6, and IL-10 have been known to have a large role in host response with respect to burns. However, there are inconsistencies as to the timing and changes of these cytokines within the literature. Most reports agree that elevations in these cytokines occur early on (hours to days) following burn injury, which is consistent with our findings found in rat burn wounds, but reports beyond this point have been variable and have been attributed to burn size, age, and even ethnicity (Jeschke et al., 2007, 2016; Finnerty et al., 2008; Coe et al., 2011; Kim et al., 2012).

The continuous inflammation of the tissue results in continuous damage that can eventually spill over into the circulatory system. Although only mild, slight elevations in WBC and DAMPs were noted for both burn depths compared to sham, while systemic cytokines showed minimal changes. Systemic changes have been correlated with burn severity, which would potentially explain why minimal changes are seen within our model, which has a 10% TBSA. Clinically, the burn patient often experiences a spike in WBC, cytokines, and DAMPs early on; however, this is typically observed in patients with >15% TBSA (Dehne et al., 2002; Lantos et al., 2010; Kim et al., 2012; Sen et al., 2019). Therefore, the 10% TBSA burn in this model may not be enough to elicit a full systemic response from burn alone. However, the slow increase in overall WBC may be a result of the sequestration of large number of inflammatory cells into the wounded tissue, which was shown by pathological assessment to be continuous throughout this study. Aside from cellular and cytokine changes, DAMPs are also well-known to result from any traumatic injury, and act as triggers to the host's innate immune machineries, which potentially can lead to subsequent adaptive immune responses. The damage associated with burn and inflammation results in DAMPs being flushed into the system from the wound, which can include HMGB-1 (Lantos et al., 2010; Huang et al., 2011), HYL (Scheibner et al., 2006), and FBN (Konter-Thioulouse et al., 1985; Sheng et al., 1987). HMGB-1 is a nuclear protein that is typically secreted by macrophages at the site of injury to attract other immune cells; however, following injury it can also be released passively by damaged/necrotic cells, such as neutrophils (Scaffidi et al., 2002). Furthermore, HMGB-1 has been positively correlated with percent TBSA, to be higher in non-surviving burn patients, and elevated in those suffering from sepsis (Lantos et al., 2010; Huang et al., 2011). HYL and FBN are both extracellular matrix components present in the skin, which are dynamically altered following tissue damage in burn patients (Konter-Thioulouse et al., 1985; Sheng et al., 1987; Onarheim et al., 1992; Scheibner et al., 2006). HYL specifically is known to fragment following burn injury and ultimately increases at systemic levels. Both HMGB-1 and HYL were seen to be elevated to some degree above the sham, as is noted to occur following burn injury (Onarheim et al., 1991, 1992; Lantos et al., 2010; Rani et al., 2017). In contrast, FBN has been shown to have the opposite effect, as compared to HMGB-1 and HYL, with levels initially decreasing post-burn and then returning to normal over the proceeding days (Lanser et al., 1980; Grzybowski et al., 1983; Konter-Thioulouse et al., 1985; Sheng et al., 1987; Thompson et al., 1989). A similar trend was seen in our rat model, with recovery of FBN levels around POD 7. Overall, the response to DPT and FT was similar, and shared some clinically relevant signatures, particularly with the local response.

Bacterial presence within any wound results in a greater challenge, as it now must contend with both the burden of initial injury and subsequent infection. In comparison to the burn-only wounds the pathologies of both DPT and FT wounds were significantly affected by the presence of *P. aeruginosa* acutely during early phases of infection followed by subsequent formation of biofilms (Brandenburg et al., 2019a,b). Pathological assessments revealed faster and greater inflammation, marked by an increased presence of degenerate/necrotic neutrophils along with macrophages. Local pro-inflammatory cytokines GRO-KC, MIP-1α, and MIP-3α were all elevated further, particularly in FT burn wounds. However, *P. aeruginosa* infection resulted in suppression of anti-inflammatory cytokines IL-10 and IL-13. Similar neutrophil and cytokine reactions have been noted to occur in other studies during acute *P. aeruginosa* infections, resulting in greater tissue damage, inflammation, and no clearance of the pathogen (Rumbaugh et al., 2001; Steinstraesser et al., 2005; Ding et al., 2015; Lin and Kazmierczak, 2017). While typical burn wounds struggle to heal due to poor orchestration of cellular and cytokine responses, the presence of *P. aeruginosa* appears to worsen the healing by inducing greater neutrophil influx, while also delaying clearance of necrotic neutrophils, which is further hampered by biofilm formation during later stages of infection (Diaz et al., 2008; Sun et al., 2012; Alhede et al., 2014; Karna et al., 2016). This continuing damage with no resolution may ultimately result in a chronic infection by *P. aeruginosa* (Bjarnsholt et al., 2008; Kirketerp-Moller et al., 2008; Fazli et al., 2009; Lin and Kazmierczak, 2017).

The local damage caused by *P. aeruginosa* eventually spills over into the circulatory system. GRO-KC and MIP-3α, which increase in the presence of microbial factors, were notably elevated at times where *P. aeruginosa* was present. Compared to the native flora, the clinical isolate of *P. aeruginosa* appears to be far more invasive, which would explain why these cytokines remained at sham levels in the burn-only controls. Our previous work also noted several cases of sepsis following FT burn injury infected with *P. aeruginosa* (Brandenburg et al., 2019a). While HYL and FBN followed similar trends to the burn-only group, HMGB-1 was further elevated by the presence of *P. aeruginosa* in the case of both burn scenarios. This could in part be due to the faster and/or greater influx of macrophages and neutrophils, which would result in greater passive and necrotic release, respectively, of HMGB-1 (Scaffidi et al., 2002). Infection driven levels of HGMB-1 have been noted to occur in burn patients, particularly for non-survivors (Huang et al., 2011). Furthermore, while FBN showed initial recovery to sham levels, infectious groups began to show signs of decline by POD 11, in the case of FT burn. Although the change is minor here, it may still be relevant as it has been noted to occur in burn patients facing infection (Lanser et al., 1980; Konter-Thioulouse et al., 1985; Sheng et al., 1987; Koenig et al., 1988). Overall, the impact of infection leads to sequelae of the burn injury, whereby causing a greater host response compared to burn alone. Longer implications can include chronic infection of the wound, systemic inflammation, multi-organ failure, sepsis, and/or death (Kallinen et al., 2012; Turner et al., 2014; Greenhalgh, 2017; Lin and Kazmierczak, 2017). Our previous work on FT burn injury with *P. aeruginosa* infection showed a septic rate of 50-100% at POD 11, depending on the starting inoculum (Brandenburg et al., 2019a). These rates are similar to those found clinically, which can range anywhere from 50 to 84%, depending on age and burn severity (Lopez et al., 2017). These results provide support for future use of this model in monitoring the effectiveness of future therapeutics to not only mitigate DAMPs, but sepsis as well.

The goal of this study was to provide a clinically relevant model of host response to acute and biofilm infection with *P. aeruginosa* in the context of burn wounds for the future use in the design and testing of therapeutics. Local inflammation related to cellular influx and cytokine levels were shown to be similar to what has been reported in clinical findings for burns and *P. aeruginosa* infections. While systemic responses were not as evident, this is likely due to the size of the burn being only 10% TBSA. Significant systemic responses typically require at least a 15–20% TBSA; therefore, the minimal systemic responses are not surprising (Walker et al., 1964; Yurt et al., 1984; Ashworth et al., 2001; Chu et al., 2005; Kim et al., 2012; Greenhalgh, 2017; Kaddoura et al., 2017). The rationale behind utilizing only a 10% TBSA model was to ensure that the rat would survive for the entirety of the longer time course study, even when exhibiting an invasive bacterial infection. Models involving larger burns (i.e., greater TBSA) and bacterial infection typically have a shorter time course or result in high mortality of the animals. A lower TBSA, coupled with the rapid diversification between rat and human immune systems, may explain the minimal effects seen in systemic responses (Weber et al., 2019). While we did see cases of sepsis, typically in the FT burns, animals survived the course of the study. One of the limitations of this model is that only minimal results concerning wound healing can be ascertained since it only extends till the very early stages of healing (if at all). Rats utilize contraction for wound closure, which typically minimizes risks of infection and/or sepsis (Abdullahi et al., 2014; Weber et al., 2019). That said, some re-epithelization and granulation tissue was noted by the pathologist, particularly in the DPT burn wounds (data not shown). Furthermore, our previous results showed that biofilm infection and/or cases of sepsis still occurred, particularly following FT burn injury, which highlights the clinical relevance of this model (Brandenburg et al., 2019a,b).

Herein, we have expanded our understanding of a clinically relevant burn model to encompass the host response to acute and biofilm infection of *P. aeruginosa* within DPT and FT burn wounds. These studies have shown that a significant local inflammatory response is generated regardless of the burn type, with such inflammation being further augmented by the presence of *P. aeruginosa*. Ultimately, this model was designed with respect to military ventures, revolving around the idea of acute care that can stabilize the wound until the injured soldier can receive proper medical attention. Having established both the infection kinetics and host response within this model, future studies will focus on therapeutics that help to mitigate infection and reduce the inflammatory state of the wound early on, with the hope of improving wound outcomes for civilians and military injuries alike.

## Data Availability Statement

All datasets generated for this study are included in the article/Supplementary Material.

## Ethics Statement

The animal study was reviewed and approved by Institutional Animal Care & Use Committee U.S. Army Institute of Surgical Research, Fort Sam Houston, TX.

## Author Contributions

AW, KB, and KL contributed conception and design of the study. AW and KB performed all animal studies, to include collection of blood, and tissue samples. BS provided pathological assessments on histology samples. AW assayed and analyzed the data, and wrote the first draft of the manuscript. All authors contributed to manuscript revision, read, and approved the submitted version.

### Conflict of Interest

The authors declare that the research was conducted in the absence of any commercial or financial relationships that could be construed as a potential conflict of interest.
