# Supplementary material for: Comparative Analysis of the Host Response in a Rat Model of Deep-Partial and Full-Thickness Burn Wounds With Pseudomonas aeruginosa Infection
Source: Front Cell Infect Microbiol. 2020 Jan 10;9:466. doi: 10.3389/fcimb.2019.00466 (PMC6967395; doi:10.3389/fcimb.2019.00466)
Supplement: Supplementary file 1 [file Table_1.docx]

**Supplemental Table 1.** Absolute values of complete blood counts for sham and burn groups. Values are presented as an average with standard deviations. White Blood Cell (WBC), Neutrophil (Neut), Lymphocyte (Lymph), Monocyte (Mono), Eosinophil (Eos), Basophil (Baso), Large Unidentified Cell (LUC).

| Sham Burn | | | | |
| --- | --- | --- | --- | --- |
| Cell Type | POD 1 | POD 3 | POD 7 | POD 11 |
| WBC [10e3/uL] | 12.24 ±1.03 | 9.3 ±1.05 | 13.38 ±3.14 | 10.24 ±1.45 |
| Neut [10e3/uL] | 3.61 ±0.47 | 2.23 ±0.41 | 2.9 ±1.35 | 1.8 ±0.99 |
| Lymph [10e3/uL] | 7.95 ±0.94 | 6.67 ±1.38 | 10.02 ±1.62 | 8.08 ±1.39 |
| Mono [10e3/uL] | 0.44 ±0.15 | 0.26 ±0.05 | 0.32 ±0.12 | 0.2 ±0.05 |
| Eos [10e3/uL] | 0.13 ±0.08 | 0.09 ±0.06 | 0.07 ±0.05 | 0.09 ±0.07 |
| Baso [10e3/uL] | 0.01 ±0.01 | 0.01 ±0.00 | 0.01 ±0.01 | 0.01 ±0.01 |
| Luc [10e3/uL] | 0.1 ±0.03 | 0.04 ±0.02 | 0.06 ±0.02 | 0.05 ±0.03 |
|  |  |  |  |  |
| DPT-Burn Only | | | | |
| Cell Type | POD 1 | POD 3 | POD 7 | POD 11 |
| WBC [10e3/uL] | 14.59 ±6.17 | 13.54 ±2.63 | 14.2 ±1.23 | 16.73 ±3.14 |
| Neut [10e3/uL] | 5.54 ±3.21 | 3.17 ±0.24 | 3.83 ±1.35 | 4.05 ±2.12 |
| Lymph [10e3/uL] | 7.83 ±2.67 | 9.6 ±2.69 | 9.03 ±0.66 | 11.26 ±1.23 |
| Mono [10e3/uL] | 0.46 ±0.15 | 0.45 ±0.18 | 0.89 ±0.46 | 0.94 ±0.29 |
| Eos [10e3/uL] | 0.09 ±0.06 | 0.06 ±0.02 | 0.09 ±0.06 | 0.05 ±0.03 |
| Baso [10e3/uL] | 0.02 ±0.02 | 0.04 ±0.02 | 0.03 ±0.01 | 0.03 ±0.02 |
| Luc [10e3/uL] | 0.64 ±0.50 | 0.22 ±0.20 | 0.33 ±0.24 | 0.4 ±0.19 |
|  |  |  |  |  |
| DPT-10^3^ Infection | | | | |
| Cell Type | POD 1 | POD 3 | POD 7 | POD 11 |
| WBC [10e3/uL] | 13.71 ±4.17 | 13.36 ±2.32 | 16.47 ±5.08 | 24.81 ±10.17 |
| Neut [10e3/uL] | 4.56 ±1.18 | 4 ±1.32 | 5 ±1.19 | 10.55 ±6.26 |
| Lymph [10e3/uL] | 8.33 ±2.93 | 8.47 ±2.75 | 9.76 ±4.17 | 12.83 ±4.12 |
| Mono [10e3/uL] | 0.42 ±0.14 | 0.55 ±0.18 | 0.71 ±0.22 | 0.81 ±0.27 |
| Eos [10e3/uL] | 0.06 ±0.01 | 0.07 ±0.07 | 0.04 ±0.02 | 0.06 ±0.03 |
| Baso [10e3/uL] | 0.02 ±0.01 | 0.03 ±0.02 | 0.05 ±0.05 | 0.08 ±0.06 |
| Luc [10e3/uL] | 0.32 ±0.22 | 0.24 ±0.16 | 0.91 ±0.67 | 0.64 ±0.39 |
|  |  |  |  |  |
| DPT-10^4^ Infection | | | | |
| Cell Type | POD 1 | POD 3 | POD 7 | POD 11 |
| WBC [10e3/uL] | 10.15 ±2.27 | 13.87 ±2.48 | 16.86 ±4.88 | 21.32 ±7.00 |
| Neut [10e3/uL] | 3.52 ±1.09 | 4.04 ±1.16 | 6.21 ±3.58 | 7.76 ±3.78 |
| Lymph [10e3/uL] | 5.93 ±1.70 | 8.95 ±2.53 | 9.06 ±3.76 | 12.03 ±3.30 |
| Mono [10e3/uL] | 0.36 ±0.08 | 0.57 ±0.20 | 0.84 ±0.35 | 1.12 ±0.56 |
| Eos [10e3/uL] | 0.03 ±0.01 | 0.08 ±0.04 | 0.1 ±0.08 | 0.04 ±0.04 |
| Baso [10e3/uL] | 0.01 ±0.00 | 0.02 ±0.02 | 0.04 ±0.02 | 0.07 ±0.03 |
| LUC [10e3/uL] | 0.31 ±0.14 | 0.22 ±0.11 | 0.61 ±0.45 | 0.29 ±0.17 |
|  |  |  |  |  |
| FT-Burn Only | | | | |
| Cell Type | POD 1 | POD 3 | POD 7 | POD 11 |
| WBC [10e3/uL] | 12.13 ±5.02 | 15.05 ±4.35 | 18.75 ±1.67 | 18.54 ±5.39 |
| Neut [10e3/uL] | 4.07 ±2.22 | 4.04 ±2.38 | 6.9 ±0.09 | 5.24 ±1.34 |
| Lymph [10e3/uL] | 7.07 ±2.33 | 10.12 ±1.95 | 9.89 ±2.02 | 11.93 ±4.21 |
| Mono [10e3/uL] | 0.41 ±0.09 | 0.48 ±0.21 | 1.01 ±0.24 | 1.01 ±0.11 |
| Eos [10e3/uL] | 0.09 ±0.03 | 0.1 ±0.04 | 0.12 ±0.02 | 0.14 ±0.08 |
| Baso [10e3/uL] | 0.02 ±0.02 | 0.03 ±0.02 | 0.04 ±0.01 | 0.03 ±0.02 |
| LUC [10e3/uL] | 0.46 ±0.41 | 0.28 ±0.15 | 0.78 ±0.56 | 0.18 ±0.10 |
|  |  |  |  |  |
| FT-10^3^ Infection | | | | |
| Cell Type | POD 1 | POD 3 | POD 7 | POD 11 |
| WBC [10e3/uL] | 12.08 ±5.66 | 14.21 ±1.74 | 16.06 ±2.62 | 19.72 ±4.99 |
| Neut [10e3/uL] | 3.9 ±1.79 | 4.64 ±1.23 | 6.1 ±1.31 | 6.73 ±2.76 |
| Lymph [10e3/uL] | 7.32 ±3.65 | 8.39 ±1.67 | 7.75 ±1.83 | 11.85 ±2.92 |
| Mono [10e3/uL] | 0.38 ±0.16 | 0.53 ±0.11 | 0.81 ±0.29 | 0.76 ±0.25 |
| Eos [10e3/uL] | 0.07 ±0.03 | 0.08 ±0.03 | 0.17 ±0.07 | 0.05 ±0.03 |
| Baso [10e3/uL] | 0.02 ±0.02 | 0.03 ±0.01 | 0.04 ±0.01 | 0.05 ±0.02 |
| LUC [10e3/uL] | 0.4 ±0.28 | 0.54 ±0.71 | 1.19 ±1.08 | 0.27 ±0.28 |
|  |  |  |  |  |
| FT-10^4^ Infection | | | | |
| Cell Type | POD 1 | POD 3 | POD 7 | POD 11 |
| WBC [10e3/uL] | 12.21 ±3.99 | 11.37 ±3.20 | 13.96 ±6.01 | 39.91 ±22.09 |
| Neut [10e3/uL] | 4.85 ±1.60 | 2.91 ±0.77 | 6.02 ±2.62 | 20.89 ±14.27 |
| Lymph [10e3/uL] | 6.35 ±2.50 | 6.46 ±2.96 | 4.75 ±2.03 | 14.31 ±9.11 |
| Mono [10e3/uL] | 0.39 ±0.18 | 0.47 ±0.37 | 0.92 ±1.26 | 1.97 ±1.10 |
| Eos [10e3/uL] | 0.05 ±0.02 | 0.02 ±0.01 | 0.04 ±0.03 | 0.04 ±0.01 |
| Baso [10e3/uL] | 0.02 ±0.01 | 0.02 ±0.01 | 0.04 ±0.03 | 0.13 ±0.11 |
| LUC [10e3/uL] | 0.56 ±0.25 | 1.48 ±0.98 | 2.18 ±1.57 | 2.58 ±3.20 |

**Supplemental Figure 1.** Comparison of local cytokine & chemokines between sham and burn-only group in DPT & FT burn wounds. Note that all cytokines & chemokines were elevated following burn injury with the exception of MIP-3α in FT injury, relative to sham. Gray, dark gray, and black bars represent the interquartile ranges of Sham Burn, DPT Burn-Only, and FT Burn-Only, respectively. ^#^*p < 0.05*, ^##^*p < 0.01*, ^###^*p < 0.001* relative to DPT burn-only. **p < 0.05* relative to FT burn-only.

 **Supplemental Figure 2.** DAMPs comparison between sham & burn groups. Burn and infection induced increased levels of HMGB-1. HYL only appeared to be influenced in the case of FT injury, whereas FBN levels appeared to return to normal in both types of burn. *P. aeruginosa* may induce reductions in FBN in the case of FT injury at POD 11. Gray, dark gray, and black bars represent the interquartile ranges of Sham Burn, DPT Burn-Only, and FT Burn-Only, respectively. ^#^*p < 0.05*, ^##^*p < 0.01* relative to DPT burn-only. **p < 0.05* relative to FT burn-only.

**Supplemental Figure 3.** Systemic cytokines and chemokines following DPT & FT injury compared to sham. Minimal changes were seen at the systemic level relative to sham. GRO/KC and MIP-3α were noted to be elevated in the presence of *P. aeruginosa*. Open squares represent sham group. Open and closed points represent DPT and FT burn wounds, respectively. Gray, dark gray, and black bars represent the interquartile ranges of burn-only, 10^3^ inoculum, and 10^4^ inoculum, respectively. ^#^*p < 0.05*, ^##^*p < 0.01* relative to DPT burn-only. **p < 0.05* relative to FT burn-only.
